# Functional Ovarian Cysts in Artificial Frozen-Thawed Embryo Transfer Cycles With Depot Gonadotropin-Releasing Hormone Agonist

**DOI:** 10.3389/fendo.2022.828993

**Published:** 2022-04-29

**Authors:** Hui Ji, Yan Su, Mianqiu Zhang, Xin Li, Xiuling Li, Hui Ding, Li Dong, Shanren Cao, Chun Zhao, Junqiang Zhang, Rong Shen, Xiufeng Ling

**Affiliations:** ^1^ Department of Reproductive Medicine, Women’s Hospital of Nanjing Medical University, Nanjing Maternity and Child Health Care Hospital, Nanjing, China; ^2^ The Fourth School of Clinical Medicine, Nanjing Medical University, Nanjing, China

**Keywords:** frozen-thawed embryo transfer, artificial cycle, functional ovarian cyst, gonadotropin-releasing hormone agonist, pregnancy outcome

## Abstract

**Objectives:**

To investigate the incidence of functional ovarian cysts, its influence on clinical rates, and proper management after depot gonadotropin-releasing hormone (GnRH) agonist pretreatment in artificial frozen-thawed embryo transfer cycles (AC-FET).

**Methods:**

This retrospective cohort study involved 3375 AC-FET cycles with follicular-phase depot GnRH agonist administration between January 2017 and December 2020. Subjects were divided into a study group (cycles with cyst formation) and a control group (cycles without cyst formation). The study group was matched by propensity scoring matching with the control group at a ratio of 1:2. For patients with ovarian cyst formation, two major managements were used: a conservative approach (i.e., expectant treatment) and a drug approach (i.e., continued agonist administration). The primary outcome was live birth rate (LBR).

**Results:**

The incidence of functional ovarian cysts following pituitary downregulation is 10.1% (341/3375). The study group exhibited a LBR similar to the control group (54.5% *vs*. 50.1%, adjusted odds ratio [aOR] 1.17, 95% confidence interval [CI] 0.88–1.56, *P =* 0.274). Patients with a lower body mass index and anti-Müllerian hormone, and a higher basal estradiol level were more susceptible to developing functional ovarian cysts. The LBR decreased after the drug approach compared with the conservative approach, but not significantly (aOR 0.63, 95% CI 0.35–1.14, *P =* 0.125). Following the conservative approach, cycles arrived at live births had a significantly shorter duration from the detection of functional cysts to the start of endometrium preparation (15.7 ± 5.1 days *vs*. 17.4 ± 5.3 days, *P* = 0.009) and a significantly higher proportion of ovarian cysts on the initial day of exogenous hormone supplementation (51.4% *vs*. 30.3%, *P* = 0.001). After controlling for all confounders, the differences remained statistically significant.

**Conclusions:**

It is unnecessary to cancel cycles that experience functional ovarian cyst formation. Conservative management and further agonist suppression protocol had similar pregnancy rates. However, a conservative approach was recommended due to its lower cost and fewer side effects. Our findings support a shorter waiting period when choosing the conservative protocol.

## Introduction

The key step in frozen-thawed embryo transfer (FET) treatment is preparing good-receptivity endometrium. Among all the FET cycle regimens, artificial cycles (AC) administer exogenous estrogen and progesterone to mimic natural cycles but do not always guarantee complete pituitary suppression (1). GnRH agonists are introduced in artificial FET cycles (AC-FET) mainly to suppress gonadotropin secretion, avoid spontaneous ovulation, and reduce cycle cancellation (2). Consequently, AC-FET protocols with gonadotropin-releasing hormone (GnRH) agonists offer the most control over cycle timing and provide less monitoring than other endometrial preparation regimens. Nevertheless, these cycles are more expensive and can have adverse effects. Current literature does not have sufficient evidence to support the use of agonist in AC-FET treatment (3).

Despite the disadvantages, a recent randomized controlled study suggested that a depot GnRH agonist applied in the follicular phase improves the implantation rate and pregnancy rates by enhancing endometrial receptivity (4). In addition, the pregnancy rates increased in women diagnosed with endometriosis (5, 6) or adenomyosis (7, 8) after GnRH agonist application before IVF or intracytoplasmic sperm injection. Irrespective of the two diseases, previous studies have shown that GnRH agonist pretreatment could increase pregnancy rates in women with polycystic ovary syndrome (PCOS) (9, 10) or repeated implantation failure (11, 12). All these findings show that agonist pretreatment is a favorable and feasible option in AC-FET cycles.

Applying a GnRH agonist can lead to a functional ovarian cyst during the treatment. No consensus has been reached yet on the exact mechanism of ovarian cyst formation. Firouzabadi et al. (13) have addressed several possible rationales: the effect of primary flare-up induced by the agonists affecting gonadotropins; inadequate inhibition of circulating gonadotropins following pituitary suppression; the direct effect of agonist on the ovaries and subsequent steroidogenesis; the quantity of progesterone at the time of agonist administration. Although such ovarian cysts have been constantly discussed in controlled ovarian stimulation (COS), there are no data on their influence on FET outcomes or what should be done when encountering these cysts.

For patients undergoing COS procedures, the ovarian cyst formation rate ranges from 5.5% (14) to 52.9% (15), mainly due to different definitions of functional cyst, administration timing, and agonist type and dose. Several publications have considered it a frustrating event that results in lower IVF outcomes (16–18). However, some authors have found that ovarian cysts after downregulation do not negatively impact the ensuing pregnancy rates (19, 20). In addition, three major managements have encountered this undesired event: cyst aspiration, continuous use of a GnRH agonist, and conservative treatment until the cyst has resolved. Still, no consensus has been reached on the optimal or proper protocol for an unexpected ovarian cyst.

Of particular interest, we conducted this retrospective study to determine the incidence and effect of a functional ovarian cyst on FET outcomes following GnRH agonist administration and provide new evidence for IVF providers and infertility couples in the management of this specific event.

## Materials and Methods

### Study Population

This retrospective cohort study was conducted at the reproductive center of Women’s Hospital of Nanjing Medical University from January 2017 to December 2020. Participants younger than 41 years who underwent AC-FET with GnRH agonist pretreatment were recruited. The exclusion criteria were: uterus malformation, fallopian hydrosalpinx, endometrial thickness (EMT) < 6 mm on the initial day of progesterone (P) exposure (21), cycles of preimplantation genetic testing, oocyte donation or vitrified oocyte, and incomplete cycle data.

### Endometrial Preparation

All FETs were performed in an artificial cycle with depot GnRH agonist downregulation (22). A baseline ultrasound was performed to detect any sign of an ovarian cyst on the second or third day of the natural or progestin-induced menstrual cycle. After excluding the presence of a functional ovarian cyst, women were intramuscularly administered a dose of 1.875 mg long-acting GnRH agonist (Diphereline, Ipsen Pharma Biotech, Signes, France). We revaluated serum hormone levels (FSH, follicle-stimulating hormone; LH, luteinizing hormone; E_2_, estradiol) and transvaginal ultrasonography 14 days later. Once pituitary downregulation criteria were achieved (EMT ≤ 5 mm, FSH < 5 mIU/mL, LH < 5 mIU/mL, and E_2_ < 50 pg/ml), the participants took 4–6 mg oral estrogen (estradiol valerate, Progynova, DELPHARM Lille SAS., Lille, France) per day for one week. Some cycles were detected with functional ovarian cysts, defined as serum E_2_ levels ≥ 50 pg/ml and a mean cyst diameter ≥ 15 mm (23). Different strategies were used to deal with these ovarian cysts, including expectant management (a conservative approach), continued GnRH agonist suppression (a drug approach), or transvaginal cyst aspiration (a surgical approach). The decision was based on the patient’s medical history and the doctor’s preference. When applying the conservative approach, some doctors preferred full resolution of ovarian cysts (cyst mean diameter < 10 mm) and fulfillment of downregulation criteria before exogenous hormone supplementation. At the same time, other clinicians waited until the downregulation criteria were achieved. Then, 4–6 mg exogenous estrogen was administered daily for a week. Regarding the drug approach, patients were administered another half or a whole shot of the agonist and carefully monitored. Since dealing with an ovarian cyst is obscure in the current literature, the decision of a half or a whole shot was based on the choice of both clinicians and patients. If patients want to have ET earlier or doctors manage things more conservatively, a half shot of agonist was administered and patients returned to the hospital 14 days later. Otherwise, patients were applied with a whole shot and reevaluated 28 days thereafter. If the baseline hormone levels were normal and the cyst did not persist, 4–6 mg/d oral estrogen was started for one week. The same estrogen dose was commenced on the second or third day of bleeding after aspiration in patients who underwent surgical aspiration.

The estrogen dose was adjusted to 6–12 mg once a day according to the EMT and serum E_2_ level in all FET cycles. After adequate endometrial proliferation with an EMT ≥ 7 mm and a serum E_2_ concentration ≥ 200 pg/ml, along with a serum level of P ≤ 1.5 ng/ml, luteal phase support (LPS) was initiated *via* vaginal administration of 90 mg progesterone (Crinone 8% gel, Fleet Laboratories Ltd., Watford, United Kingdom) once and 10 mg of dydrogesterone (Abbott Biologicals B.V., Weesp, the Netherlands) thrice every day. A total of 21 patients could only achieve a maximum EMT between ≥6mm and <7mm after long estrogen exposure but was the thickest EMT they could achieve. Such cycles were included in the final analysis, accounting for about 2.2% of the whole population (22/935). Cycles were canceled if the serum P level was >1.5 ng/ml prior to LPS, or a prolonged period of estrogen priming (more than 24 days) was required. In the case of pregnancy, LPS was continued until 10–12 weeks of gestation.

### Blastocyst Grading

After surviving the thawing procedure, day (D) 3 cleavage-stage embryos were cultured for another 16 hours before the transfer based on our work schedule. For D5 or D6 blastocysts, additional 2–6 h incubation was performed before transfer. Cryopreserved cleavage-stage embryos or blastocysts were transferred 3 and 5 days after progesterone initiation, respectively. The D3 embryos reaching the morula stage which containing 16–32 blastomeres with > 90% of its cell mass compacted were good-quality embryos (24). According to Gardner’s scoring system, the blastocysts were graded on the basis of three parameters: cavity expansion, inner cell mass (ICM), and trophectoderm (TE) (25). Each blastocyst was scored according to the degree of cavity expansion to obtain 1–6 grades. Once the embryo reached the expansion level 3 or above, ICM and TE were graded according to the cell size and density (A, B, or C). A blastocyst graded ≥ 3 with A or B for both ICM and TE was defined as a good-quality blastocyst (grades 3–6 AA/AB/BA/BB); otherwise, it was considered low quality.

### Pregnancy Outcome

The primary outcome of this study was the live birth rate (LBR). Live birth was defined as the delivery of a viable infant after 28 weeks of gestation. The secondary outcomes included the implantation rate (IR), clinical pregnancy rate (CPR), and abortion rate (AR). The IR was calculated from the number of gestational sacs per number of transferred embryos. The clinical pregnancy was determined by gestational sac ultrasound at 6–7 weeks of gestation. Abortion was defined as a pregnancy loss during the first and second trimesters.

### Statistical Analysis

All data were analyzed using the SPSS software version 26 (IBM Corp., Armonk, NY, USA). We used the Student’s *t*-test or Mann-Whitney U test (if data were not normally distributed) for quantitative variables and Pearson’s χ2 test or Fisher’s exact test for categorical variables. To compare pregnancy rates between the cyst-positive and cyst-negative groups, we used the propensity scoring matching (PSM) method to alleviate potential selection bias. The final variables included in the PSM analysis model were patient age, infertility type, duration and cause, body mass index (BMI), baseline FSH and anti-Müllerian hormone (AMH) levels, EMT, embryo developmental stage, number of transferred embryos, and good-quality embryo number. After calculating the propensity score of each subject, patients in the cyst-positive group were matched in a 1:2 ratio with patients in the cyst-negative group with a 0.1 caliper width using the nearest neighbor matching. Moreover, multivariate regression analysis was conducted to identify the factors that had a significant effect on the occurrence of functional cysts. The multiple regression model contained variables that showed significant differences on univariate analysis at *P* < 0.1. Additionally, the confounders with a *P*-value < 0.1 or had a significant influence on LBR were included in the multivariable logistic regression to estimate the independent effect of ovarian cyst formation and different solutions on LBR.

Continuous data are presented as the mean ± SD following the *t*-test and median (Inter-Quartile Range, IQR) derived from the U test. Results were expressed as the adjusted odds ratio (aOR) and 95% confidence intervals (95% CI) in the multivariate regression analysis. The statistical significance was accepted at *P*-value < 0.05.

## Results

**Figure 1:**
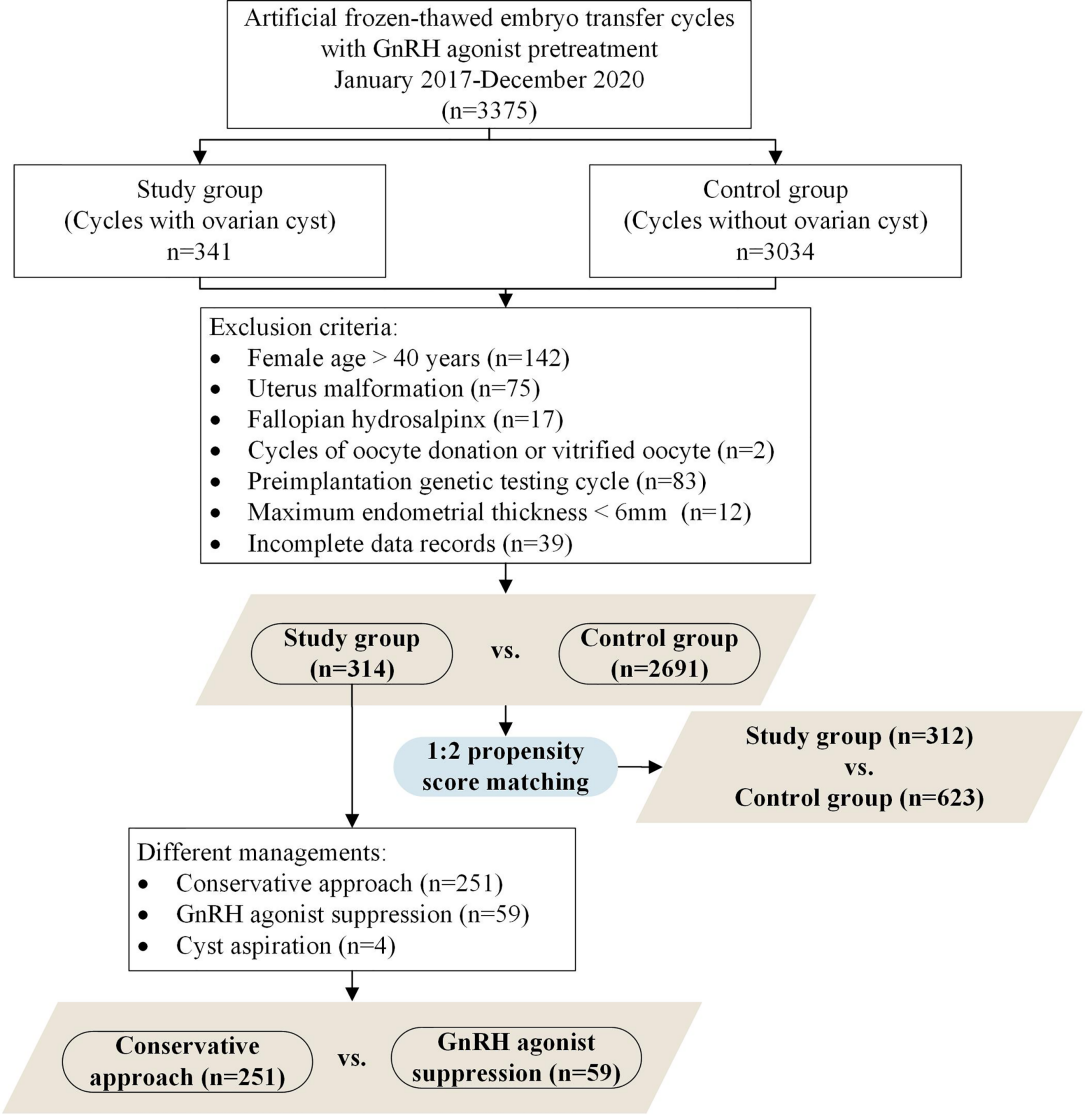
Flow chart.

The patient selection flow chart is shown in **Figure 1**. Initially, we conducted 3375 frozen-thawed autologous embryo transfer cycles using downregulation protocol between January 01, 2017, and December 31, 2020. The FET cycles were divided into two groups according to the occurrence of functional ovarian cysts after GnRH agonist: the study group (cycles with ovarian cyst formation, n = 341) and the control group (cycles without ovarian cyst formation, n = 3034). After excluding 370 cycles, a total of 3005 FET cycles were included (314 cycles in the study group and 2691 in the control group). The number of participants after PSM in the study and control groups was 312 and 623, respectively.

The demographic parameters of the unpaired and paired participants in the two groups are summarized in **Table 1**. There were significant differences in terms of BMI, serum levels of basal E_2_ and AMH, and EMT within groups before PSM analysis (*P* < 0.01). After PSM, the demographics of the matched groups were comparable (*P* > 0.05).

**Table 1:** Demographic characteristics before and after propensity score matching.

	Before PSM			After PSM		
Characteristic	Study group	Control group	*P* value	Study group	Control group	*P* value
	(n=314)	(n=2691)		(n=312)	(n=623)	
Age (years)	30.3 ± 4.0	30.2 ± 4.0	0.842	30.2 ± 3.9	30.4 ± 4.0	0.616
Infertility type, n (%)			0.654			0.418
primary	178 (56.7)	1561 (58.0)		177 (56.7)	336 (53.9)	
secondary	136 (43.3)	1130 (42.0)		135 (43.3)	287 (46.1)	
Duration of infertility, n (%)			0.355			0.449
1-2	92 (29.3)	740 (27.5)		92 (29.5)	172 (27.6)	
3-5	185 (58.9)	1555 (57.8)		184 (59.0)	361 (57.9)	
≥6	37 (11.8)	396 (14.7)		36 (11.5)	90 (14.4)	
Cause of infertility, n (%)			0.265			0.869
female	148 (47.1)	1387 (51.5)		146 (46.8)	309 (49.6)	
male	23 (7.3)	202 (7.5)		23 (7.4)	46 (7.4)	
combined	118 (37.6)	949 (35.3)		118 (37.8)	222 (35.6)	
unknown	25 (8.0)	153 (5.7)		25 (8.0)	46 (7.4)	
BMI (kg/m^2^)	21.2 ± 2.8	22.7 ± 3.4	<0.001	21.3 ± 2.8	21.2 ± 2.8	0.930
Basal FSH (mIU/mL)	7.7 ± 2.2	7.5 ± 2.5	0.164	7.7 ± 2.2	7.7 ± 2.4	0.626
Basal E_2_ (pg/ml)	50.9 ± 23.8	46.0 ± 20.5	<0.001	50.5 ± 23.2	51.2 ± 22.3	0.620
AMH (ng/ml)*	5.0 (3.0-7.9)	5.6 (3.0-9.9)	0.014	5.0 (3.1-7.9)	5.1 (2.8-9.2)	0.610
Endometrial thickness (mm)	9.9 ± 1.8	10.2 ± 1.9	0.004	9.9 ± 1.8	9.8 ± 1.8	0.497
Embryo developmental stage, n (%)			0.656			0.948
D3	100 (31.8)	824 (30.6)		100 (32.1)	201 (32.3)	
D5/D6	214 (68.2)	1867 (69.4)		212 (67.9)	422 (67.7)	
Number of transferred embryos	1.8 ± 0.4	1.8 ± 0.5	0.825	1.8 ± 0.4	1.8 ± 0.5	0.264
Number of good-quality embryos			0.467			0.984
0	103 (32.8)	927 (34.4)		103 (33.0)	205 (32.9)	
1	137 (43.6)	1078 (40.1)		135 (43.3)	273 (43.8)	
2	74 (23.6)	686 (25.5)		74 (23.7)	145 (23.3)	

*Data are presented as the median (quartiles). Other data are presented as mean ± SD or n (%). PSM, propensity score matching; BMI, body mass index; FSH, follicle-stimulating hormone; E2, estradiol; AMH, anti-Müllerian hormone.

We applied the multivariable logistic regression analysis to predict the possible confounders for the formation of ovarian cysts (**Table 2**). Patients with a higher BMI (aOR 0.87, 95% CI 0.84–0.91, *P* < 0.001) and AMH value (aOR 0.97, 95% CI 0.95–0.99, *P* = 0.007) were less susceptible to ovarian cysts after GnRH agonist supplementation, whereas a higher basal E_2_ level increased their incidence (aOR 1.01, 95% CI 1.00–1.01, *P* = 0.002).

**Table 2:** Variables associated with the functional ovarian cyst formation in artificial frozen-thawed embryo transfer cycles with GnRH agonist: multivariate logistic regression analysis.

	aOR	95% CI	*P* value
BMI	0.87	0.84-0.91	<0.001
Basal E_2_	1.01	1.00-1.01	0.002
AMH	0.97	0.95-0.99	0.007

The multiple regression model included variables that showed significant differences on univariate analysis at P < 0.1.

Clinical outcomes after PSM are shown in **Table 3**. The IR (44.4% *vs*. 41.2%, *P* = 0.217), CPR (60.3% *vs*. 58.1%, *P* = 0.529), AR (9.6% *vs*. 13.8%, *P =* 0.152), and LBR (54.5% *vs*. 50.1%, *P* = 0.204) were comparable in the study group than those in the control group. We further analyzed the influence of functional cysts on LBR in the multivariate logistic regression. An initial cohort of 935 FET cycles was divided into two groups: the LB group (cycles reaching live births) and the non-LB group (cycles failing to have live births) (**Table 4**). FET cycles with ovarian cyst formation had a higher LBR than those without ovarian cyst, but with no statistical difference (aOR 1.17, 95% CI 0.88–1.56, *P* = 0.274) (**Table 5**).

**Table 3:** Clinical outcomes after propensity score matching.

Outcome	Study group	Control group	*P* value
	(n=312)	(n=623)	
Implantation rate, n/N (%)	249/561 (44.4)	453/1099 (41.2)	0.217
Clinical pregnancy rate, n/N (%)	188/312 (60.3)	362/623 (58.1)	0.529
Abortion rate, n/N (%)	18/188 (9.6)	50/362 (13.8)	0.152
Live birth rate, n/N (%)	170/312 (54.5)	312/623 (50.1)	0.204

**Table 4:** Characteristics between cycles reaching live births or failing to have live births in the overall population after propensity score matching.

Characteristic	LB group	Non-LB group	*P* value
	(n=482)	(n=453)	
Age (years)	29.9 ± 3.7	30.8 ± 4.2	<0.001
Infertility type, n (%)			0.075
primary	278 (57.5)	235 (51.9)	
secondary	204 (42.3)	218 (48.1)	
Duration of infertility, n (%)			0.729
1-2	139 (28.8)	125 (27.6)	
3-5	282 (58.5)	263 (58.1)	
≥6	61 (12.7)	65 (14.3)	
Cause of infertility, n (%)			0.513
female	233 (48.3)	222 (49.0)	
male	37 (7.7)	32 (7.1)	
combined	181 (37.6)	159 (35.1)	
unknown	31 (6.4)	40 (8.8)	
BMI (kg/m^2^)	21.2 ± 2.9	21.3 ± 2.8	0.843
Basal FSH (mIU/mL)	7.5 ± 2.1	7.9 ± 2.6	0.025
Basal E_2_ (pg/ml)	51.0 ± 22.9	51.0 ± 22.2	0.984
AMH (ng/ml)	5.3 (3.4-8.7)	4.4 (2.5-8.5)	<0.001
Endometrial thickness (mm)	9.9 ± 1.7	9.7 ± 1.9	0.127
Embryo developmental stage, n (%)			<0.001
D3	121 (25.1)	180 (39.7)	
D5/D6	361 (74.9)	273 (60.3)	
Number of transferred embryos	1.8 ± 0.4	1.8 ± 0.5	0.052
Number of good-quality embryos			<0.001
0	113 (23.4)	195 (43.0)	
1	227 (47.1)	181 (40.0)	
2	142 (29.5)	77 (17.0)	
Ovarian cysts formation			0.204
no	312 (64.7)	311 (68.7)	
yes	170 (35.5)	142 (31.3)	

LB, live birth.

**Table 5:** Variables associated with live birth rate: multivariate logistic regression analysis.

	aOR	95% CI	*P* value
**Formation of functional ovarian cyst ****^a^**			
no	Reference		
yes	1.17	0.88-1.56	0.274
**Different approach managing ovarian cyst ****^b^**			
conservative approach	Reference		
drug approach	0.63	0.35-1.14	0.125
**Confounders in the conservative approach ****^c^**			
duration between cyst detection and estrogen application	0.94	0.89-0.99	0.025
ovarian cyst on estrogen starting day			
no	Reference		
yes	2.11	1.19-3.74	0.010

a : Adjusted for maternal age, infertility type, BMI, AMH, endometrial thickness, embryo developmental stage, number of transferred embryos, number of good-quality embryos, and formation of functional ovarian cyst (yes vs. no).

b : Adjusted for maternal age, BMI, AMH, endometrial thickness, embryo developmental stage, number of transferred embryos, number of good-quality embryos, and different approach managing ovarian cyst (continued agonist suppression vs. conservative approach).

c : Adjusted for maternal age, infertility type, BMI, AMH, endometrial thickness, embryo developmental stage, number of transferred embryos, number of good-quality embryos, duration between cyst detection and estrogen supplementation, and presence of ovarian cyst on estrogen supplementation day (yes vs. no).

A total of 314 patients had functional cysts after pituitary suppression and were treated with three different protocols: the conservative approach (n = 251), the drug approach (n = 59), and the surgical approach (n = 4). Due to the small sample size of cyst aspiration, we only analyzed the effectiveness of the conservative versus the drug approach. The baseline demographic characteristics of the two cohorts are listed in **Table 6**. The LB group yielded a higher proportion of the conservative approach than the non-LB group, although the difference was not statistically significant (84.5% *vs*. 76.8%, *P* = 0.083). With the different strategies as the main exposure of interest, the logistic regression analysis revealed that the drug approach had no statistically negative influence on LBR compared with the conservative protocol (aOR 0.63, 95% CI 0.35–1.14, *P* = 0.125) (**Table 5**).

**Table 6:** Characteristics between cycles reaching live births or failing to have live births under conservative and drug approach.

Characteristic	LB group	Non-LB group	*P* value
	(n=168)	(n=142)	
Age (years)	30.0 ± 3.7	30.6 ± 4.1	0.155
Infertility type, n (%)			0.100
primary	102 (60.7)	73 (51.4)	
secondary	66 (39.3)	69 (48.6)	
Duration of infertility, n (%)			0.930
1-2	50 (29.8)	41 (28.9)	
3-5	99 (58.9)	83 (58.5)	
≥6	19 (11.3)	18 (12.7)	
Cause of infertility, n (%)			0.359
female	80 (47.6)	68 (47.9)	
male	13 (7.7)	10 (7.0)	
combined	66 (39.3)	49 (34.5)	
unknown	9 (5.4)	15 (10.6)	
BMI (kg/m^2^)	21.3 ± 3.1	21.2 ± 2.6	0.962
Basal FSH (mIU/mL)	7.6 ± 2.2	7.8 ± 2.2	0.409
Basal E_2_ (pg/ml)	49.0 ± 22.3	53.3 ± 25.5	0.115
AMH (ng/ml)	5.2 (3.4-8.2)	4.2 (2.9-8.4)	0.117
Endometrial thickness (mm)	10.0 ± 1.7	9.8 ± 2.0	0.265
Embryo developmental stage, n (%)			0.106
D3	46 (27.4)	51 (35.9)	
D5/D6	122 (72.6)	91 (64.1)	
Number of transferred embryos	1.8 ± 0.4	1.7 ± 0.5	0.048
Number of good-quality embryos			0.025
0	50 (29.8)	53 (37.3)	
1	69 (41.1)	66 (46.5)	
2	49 (29.2)	23 (16.2)	
Ovarian cysts			0.083
conservative approach	142 (84.5)	109 (76.8)	
drug approach	26 (15.5)	33 (23.2)	
Number of ovarian cysts			0.775
1	82 (48.8)	67 (47.2)	
≥2	86 (51.2)	75 (52.8)	
Ovarian cysts			0.140
unilateral	113 (67.3)	84 (59.2)	
bilateral	55 (32.7)	58 (40.8)	
Largest cyst diameter (mm)	25.9 ± 6.1	27.0 ± 7.0	0.149

If we focused on live births only in the drug approach, the LBR was associated with a shorter waiting period between the detection of functional cysts and the start of endometrium preparation (15.7 ± 5.1 days *vs*. 17.4 ± 5.3 days, aOR 0.94, 95% CI 0.89–0.99, *P* = 0.025) and a higher rate of ovarian cysts on estrogen starting day (51.4% *vs*. 30.3%, aOR 2.11, 95% CI 1.19–3.74, *P* = 0.010), both in univariate and crude analysis (**Tables 5**, **7**).

**Table 7:** Characteristics between cycles reaching live births or failing to have live births under the conservative approach.

Characteristic	LB group	Non-LB group	*P* value
	(n=142)	(n=109)	
Age (years)	29.7 ± 3.5	30.6 ± 4.1	0.059
Infertility type, n (%)			0.081
primary	86 (60.6)	54 (49.5)	
secondary	56 (39.4)	55 (50.5)	
Duration of infertility, n (%)			0.827
1-2	44 (31.0)	30 (27.5)	
3-5	81 (57.0)	66 (60.6)	
≥6	17 (12.0)	13 (11.9)	
Cause of infertility, n (%)			0.333
female	69 (48.6)	48 (44.0)	
male	9 (6.3)	8 (7.3)	
combined	56 (39.4)	40 (36.7)	
unknown	8 (5.6)	13 (11.9)	
BMI (kg/m^2^)	21.2 ± 3.2	21.2 ± 2.6	0.969
Basal FSH (mIU/mL)	7.7 ± 2.2	7.7 ± 2.0	0.788
Basal E_2_ (pg/ml)	47.3 ± 20.4	51.7 ± 23.8	0.120
AMH (ng/ml)	5.2 (3.4-8.2)	4.2 (2.9-8.4)	0.117
Endometrial thickness (mm)	10.0 ± 1.7	9.8 ± 2.0	0.458
Embryo developmental stage, n (%)			0.050
D3	37 (26.1)	41 (37.6)	
D5/D6	105 (73.9)	68 (62.4)	
Number of transferred embryos	1.8 ± 0.4	1.7 ± 0.5	0.041
Number of good-quality embryos			0.072
0	43 (30.3)	41 (37.6)	
1	55 (38.7)	48 (44.0)	
2	44 (31.0)	20 (18.3)	
Duration between cyst detection and estrogen application (days)	15.7 ± 5.1	17.4 ± 5.3	0.009
Ovarian cyst on estrogen starting day			0.001
no	69 (48.6)	76 (69.7)	
yes	73 (51.4)	33 (30.3)	

## Discussion

One side effect of AC-FET cycles with GnRH agonist co-treatment is the unexpected formation of functional ovarian cysts. Although their incidence and management have raised constant debate in the COS process, there is a lack of data regarding the event’s influence on pregnancy outcomes in FET cycles. Our findings confirmed that the LBR was not compromised in FET cycles with ovarian cysts. Neither conservative management nor further agonist suppression improved clinical outcomes. However, a conservative approach was recommended due to its lower cost and fewer side effects. Data also supports a shorter waiting period when choosing the conservative protocol.

In this study, the formation rate of functional ovarian cysts was 10.1% (341/3375) in patients treated with follicular-phase depot GnRH agonist, similar to the 9.3% after the luteal-phase suppression as reported by Qublan et al. (23). Other investigators found that agonist injection in the follicular phase was associated with a higher rate of cyst formation (14, 26). However, their findings contradict a previous study in which the event incidence was higher when administered at mid-luteal phase than menstruation (15.4% *vs*. 13.6%) (27). Meanwhile, the occurrence of the ovarian cyst had no apparent connection with the type or route of a specific agonist when injected at menstruation (28). In general, cyst formation will always happen no matter what, when, or how the agonist is used. In that case, it is logical and meaningful to focus on this specific issue.

Cyst formation does not happen at random and might indicate patients with a poor ovarian response according to published data (13, 16, 17, 23). The levels of lower AMH correlating with higher basal E_2_ are significant predictors for poor ovarian reserve (29, 30). Additionally, E_2_ increases pituitary sensitivity to GnRH by stimulating an increase in the expression of the gene encoding the GnRH receptor (31). Due to the primary flare-up caused by the agonist, a relatively high serum E_2_ and low AMH may thus have a promoted effect on the induction of cyst formation. Our results concur with findings that ovarian cysts were significantly associated with patients with higher basal E_2_ and lower AMH levels. However, none of these papers reported patient BMI as part of their data. Women diagnosed with PCOS are considered high ovarian responders (32) and generally display a higher BMI than normal population (33–35). This finding would partially explain why participants with a higher BMI in our study are less likely to have functional cysts; however, the underlying biological mechanisms require further study.

The effect of the ovarian cyst presence on pregnancy outcomes has been discussed frequently in COS treatment and fresh ET cycles, but the findings are conflicting. Some investigations point out that the existence of ovarian cysts increased the cycle cancellation rate, lowered oocyte number and quality, and compromised the pregnancy results (14, 16, 23, 36). In such cases, the cysts inside the ovary would impede the final stages of the pre-ovulatory follicles, reduce the space for other follicles to develop, and damage the blood supply to the growing follicles (14, 36), all contributing to poor oocyte results. However, other studies failed to show any significant difference between cyst-positive and cyst-negative cycles after GnRH agonist therapy (27, 37). Our results demonstrate that functional cysts have no significant detrimental effect on LBR following the transfer of vitrified embryos (54.5% *vs*. 50.1%, *P* = 0.204). Since the major focus is endometrium growth rather than follicle development in FET cycles, it is understandable that patients with functional cysts do not experience a decline in clinical rates.

Different approaches have been used in managing these functional cysts following GnRH agonist administration. The most beneficial protocol before COS in IVF cycles has been the subject of several retrospective papers. There were comparable pregnancy rates between patients who underwent ovarian cyst aspiration and those who chose the conservative approach (13, 14, 23, 36). Considering the extra cost, additional risk of surgical complications, and emotional burden related to the surgery, a systematic review did not provide supportive evidence for cyst drainage before COS (38). In consistent with these reports, most physicians in our center do not consider cyst aspiration a patient-friendly option. Therefore, only four women underwent cyst aspiration surgery in the present study, a number too small for effective statistical analysis. Yet, in light of the absence of existing data between the effectiveness of expectant management and continuous suppression with GnRH agonist, we compared the FET outcomes between the two protocols. The LBR in the drug approach was lower than that in the conservative approach, although the difference did not reach statistical significance (aOR 0.63, 95% CI 0.35–1.14, *P* = 0.125). A tendency was observed in favor of the conservative approach than the drug approach. However, the most prominent side effect of continued depot GnRH agonist is estrogen deficiency, which will cause a menopause-like state (39). Therefore, the findings of our study recommend the conservative approach as the first choice for women with an ovarian cyst undergoing FET, particularly in terms of lower cost and more safety. Notwithstanding, one should be cautious about the interpretation of this finding because the decision-making of two available strategies is not randomly controlled and mainly at the physician’s discretion. Data supported endometriosis (40) or adenoma (41) were associated with a worse prognosis versus other infertility factors. When dealing with functional cysts in these women, doctors might continue agonist suppression at a higher odd.

More specifically, we investigated the confounders affecting LBR in patients undergoing a conservative approach. The shorter duration between the day of ovarian cyst occurrence and exogenous hormone initiation, and the presence of persistent cysts are both protective factors for live births. The data suggest that there may be an advantage in shortening the waiting period instead of considering a fully diminished follicular cyst. Once the serum hormone levels and EMT reached the downregulation criteria, exogenous estrogen could be initiated to proliferate the endometrium to achieve optimal outcomes. This referred result is in line with Segal et al. (18) and Zeyneloglu et al. (42), revealing that longer suppression with a GnRH agonist ended with lower pregnancy rates.

The body of literature indicates that a half-dose injection of depot GnRH agonist (1.875 mg) is equally effective for pituitary desensitization compared with a full-dose (3.75 mg) or a long multiple-dose (43–45). Furthermore, pituitary desensitization usually occurs approximately 14 days after agonist supplementation and continues until the eighth week after the injection (46). The depot GnRH agonist administration in our study was used at a dose of 1.875 mg during menstruation, and the hormone levels and ultrasound were reassessed in the subsequent two weeks. The incidence is about 10.1%, which is not a rare but a common event. Our study is the first to focus on the subject of functional ovarian cysts in AC-FET cycles following GnRH agonist suppression. We believe that our findings are valuable in clinical practice and could provide crucial evidence for both physicians and patients.

Despite our efforts, the present research has some limitations that need to be taken into consideration. First, it was conducted at a single institution. Second, the retrospective nature of this study and the inherent selection biases therein. For instance, the choice to proceed with the conservative protocol or further agonist administration was based on the doctor’s preference, which could interfere with the final results. Lastly, the limited sample size was not large enough to arrive at sufficiently convincing conclusions. Future studies that include a larger sample size are needed to validate the findings of this retrospective study and provide more information.

## Conclusions

In conclusion, our study suggests that functional ovarian cysts do not pose any detrimental effect on pregnancy rates following FET treatment. The prevalence of cyst formation increased with increasing basal E_2_ levels and lower AMH and BMI values. Patients who underwent a conservative approach had similar clinical outcomes than those with further agonist suppression. To avoid medical costs and potential side effects, it may be wise to conservatively treat women with ovarian cysts until further evidence is available. Under the conservative strategy, it is unnecessary to initiate exogenous estrogen administration until the cyst has fully resolved; a short waiting period can obtain better pregnancy results once downregulation has been achieved.

## Data Availability Statement

The raw data supporting the conclusions of this article will be made available by the authors, without undue reservation.

## Ethics Statement

The studies involving human participants were reviewed and approved by The Ethics Committee of Women’s Hospital of Nanjing Medical University (NJFY-2020-KY-070). The ethics committee waived the requirement of written informed consent for participation.

## Author Contributions

HJ collected data, performed the analysis, and wrote the manuscript. YS participated in the study design and drafted the article. MZ and XLi participated in the acquisition and analysis of data. XLLi, HD, LD, SC, CZ, and JZ reviewed the final article and made important intellectual contents. RS and XLing were corresponding authors and they participated in the study design, did the final proof reading and confirmed the final version. All authors contributed to the article and approved the submitted version.

## Funding

The study was funded by National Natural Science Foundation of China (grant no. 81771536, 81871210), Program for the Top Innovative Talents of “Six Major Projects” of Jiangsu Province (grant no. LGY2018004), and Open fund of State Key Laboratory of Reproductive Medicine, Nanjing Medical University (grant no. SKLRM-K201806). The open access publication fees are covered by National Natural Science Foundation of China (grant no 81871210).

## Conflict of Interest

The authors declare that the research was conducted in the absence of any commercial or financial relationships that could be construed as a potential conflict of interest.

## Publisher’s Note

All claims expressed in this article are solely those of the authors and do not necessarily represent those of their affiliated organizations, or those of the publisher, the editors and the reviewers. Any product that may be evaluated in this article, or claim that may be made by its manufacturer, is not guaranteed or endorsed by the publisher.
